# Vaccine effectiveness of two-dose BNT162b2 against symptomatic and severe COVID-19 among adolescents in Brazil and Scotland over time: a test-negative case-control study

**DOI:** 10.1016/S1473-3099(22)00451-0

**Published:** 2022-11

**Authors:** Pilar T V Florentino, Tristan Millington, Thiago Cerqueira-Silva, Chris Robertson, Vinicius de Araújo Oliveira, Juracy B S Júnior, Flávia J O Alves, Gerson O Penna, Srinivasa Vital Katikireddi, Viviane S Boaventura, Guilherme L Werneck, Neil Pearce, Colin McCowan, Christopher Sullivan, Utkarsh Agrawal, Zoe Grange, Lewis D Ritchie, Colin R Simpson, Aziz Sheikh, Mauricio L Barreto, Igor Rudan, Manoel Barral-Netto, Enny S Paixão

**Affiliations:** a Centre of Data and Knowledge Integration for Health (CIDACS), Gonçalo Moniz Institute, Oswaldo Cruz Foundation, Salvador, Brazil; b Biomedical Science Institute, University of São Paulo, São Paulo, Brazil; c Usher Institute, University of Edinburgh, Edinburgh, UK; d LIB and LEITV Laboratories, Instituto Gonçalo Moniz, Oswaldo Cruz Foundation, Salvador, Brazil; e Institute of Collective Health, Federal University of Bahia, Salvador, Brazil; f Faculty of Medicine, Federal University of Bahia, Salvador, Brazil; g Public Health Scotland, Glasgow, Scotland, UK; h Tropical Medicine Centre, University of Brasília, Fiocruz School of Government Brasília, Brasília, Brazil; i MRC/CSO Social and Public Health Sciences Unit, University of Glasgow, Glasgow, UK; j Department of Epidemiology, Social Medicine Institute, State University of Rio de Janeiro, Rio de Janeiro, Brazil; k Institute of Collective Health Studies, Federal University of Rio de Janeiro, Rio de Janeiro, Brazil; l Faculty of Epidemiology and Population Health, London School of Hygiene & Tropical Medicine, London, UK; m School of Medicine, University of St Andrews, St Andrews, Scotland, UK; n Academic Primary Care, University of Aberdeen, Aberdeen, Scotland, UK; o School of Health, Wellington Faculty of Health, Victoria University of Wellington, Wellington, New Zealand

## Abstract

**Background:**

Little is known about vaccine effectiveness over time among adolescents, especially against the SARS-CoV-2 omicron (B.1.1.529) variant. This study assessed the associations between time since two-dose vaccination with BNT162b2 and the occurrence of symptomatic SARS-CoV-2 infection and severe COVID-19 among adolescents in Brazil and Scotland.

**Methods:**

We did test-negative, case-control studies in adolescents aged 12–17 years with COVID-19-related symptoms in Brazil and Scotland. We linked records of SARS-CoV-2 RT-PCR and antigen tests to national vaccination and clinical records. We excluded tests from individuals who did not have symptoms, were vaccinated before the start of the national vaccination programme, received vaccines other than BNT162b2 or a SARS-CoV-2 booster dose of any kind, or had an interval between their first and second dose of fewer than 21 days. Additionally, we excluded negative SARS-CoV-2 tests recorded within 14 days of a previous negative test, negative tests recorded within 7 days after a positive test, any test done within 90 days after a positive test, and tests with missing sex and location information. Cases (SARS-CoV-2 test-positive adolescents) and controls (test-negative adolescents) were drawn from a sample of individuals in whom tests were collected within 10 days of symptom onset. We estimated the adjusted odds ratio and vaccine effectiveness against symptomatic COVID-19 for both countries and against severe COVID-19 (hospitalisation or death) for Brazil across fortnightly periods.

**Findings:**

We analysed 503 776 tests from 2 948 538 adolescents in Brazil between Sept 2, 2021, and April 19, 2022, and 127 168 tests from 404 673 adolescents in Scotland between Aug 6, 2021, and April 19, 2022. Vaccine effectiveness peaked at 14–27 days after the second dose in both countries during both waves, and was significantly lower against symptomatic infection during the omicron-dominant period in Brazil (64·7% [95% CI 63·0–66·3]) and in Scotland (82·6% [80·6–84·5]), than it was in the delta-dominant period (80·7% [95% CI 77·8–83·3] in Brazil and 92·8% [85·7–96·4] in Scotland). Vaccine efficacy started to decline from 27 days after the second dose for both countries, reducing to 5·9% (95% CI 2·2–9·4) in Brazil and 50·6% (42·7–57·4) in Scotland at 98 days or more during the omicron-dominant period. In Brazil, protection against severe disease remained above 80% from 28 days after the second dose and was 82·7% (95% CI 68·8–90·4) at 98 days or more after receiving the second dose.

**Interpretation:**

We found waning vaccine protection of BNT162b2 against symptomatic COVID-19 infection among adolescents in Brazil and Scotland from 27 days after the second dose. However, protection against severe COVID-19 outcomes remained high at 98 days or more after the second dose in the omicron-dominant period. Booster doses for adolescents need to be considered.

**Funding:**

UK Research and Innovation (Medical Research Council), Scottish Government, Health Data Research UK BREATHE Hub, Fiocruz, Fazer o Bem Faz Bem programme, Brazilian National Research Council, and Wellcome Trust.

**Translation:**

For the Portuguese translation of the abstract see Supplementary Materials section.

## Introduction

Clinical trials^1^ and observational studies^2^, ^3^, ^4^ of adolescents aged 12–17 years have reported that two doses of the mRNA BNT162b2 (Pfizer-BioNTech) vaccine provide substantial short-term protection against symptomatic SARS-CoV-2 infection and COVID-19 hospital admission. However, little is known about vaccine effectiveness over time in this population, especially against the SARS-CoV-2 omicron (B.1.1.529) variant.

The WHO Technical Advisory Group classified omicron as a variant of concern due to the large number of mutations in the receptor-binding domain of its spike protein that are associated with enhanced transmissibility and immune evasion.^5^ Despite increasing vaccination coverage, reports of increasing infection rates in children and adolescents over time are of concern.^6^ Although these increased infection rates might be attributable to omicron's ability to evade natural and vaccine-induced protection,^7^ it is also plausible that vaccine effectiveness wanes over time, as previously shown in the adult population.^8^ A 2022 study indicated that BNT162b2 vaccine effectiveness against COVID-19-associated visits to the emergency room among adolescents was significantly lower during the omicron period than in the period in which the delta (B.1.617.2) variant predominated.^9^ Also, no significant protection against symptomatic COVID-19 was observed 150 days or more after the second vaccine dose during the period when the omicron variant predominated in the USA.^9^ However, for hospitalisation 150 days or more after the second vaccine dose, the vaccine effectiveness was 73% (95% CI 43–88) in adolescents aged 12–15 years and 94% (87–97) in those aged 16–17 years.^9^ Nevertheless, it remains unclear how vaccine-induced protection varies over time in adolescents. This knowledge could have important policy implications (eg, for informing the need for providing booster doses in this population).

**Research in context**

**Evidence before this study**

We searched PubMed from March 1, 2022, up to June 22, 2022, for published papers using the terms “vaccine effectiveness” AND “adolescents” AND “Omicron OR B.1.1.529”. We found four observational studies reporting high vaccine effectiveness of two doses of the BNT162b2 mRNA vaccine against symptomatic infection and COVID-19-related hospitalisations among adolescents aged 12–17 years during the SARS-CoV-2 omicron (B.1.1.529)-dominant period. However, only one study done in England estimated vaccine effectiveness over time against symptomatic COVID-19, and no data so far have reported protection over time against severe outcomes in adolescents during the period in which the omicron variant was dominant.

**Added value of this study**

Our cross-country analyses in Brazil and Scotland showed that BNT162b2 vaccine effectiveness against symptomatic SARS-CoV-2 among adolescents rapidly declined over time, reaching 5·9% (95% CI 2·2–9·4) in Brazil and 50·6% (95% CI 42·7–57·4) in Scotland at 98 days or more after the second vaccine dose during the omicron-dominant period (from Jan 1, 2022, to April 19, 2022). However, protection against severe disease in Brazil after two doses of vaccine was maintained at more than 80% at 98 days or more after receiving the second dose. To our knowledge, this is the first nationwide study to evaluate vaccine effectiveness over time against severe COVID-19 among adolescents during the omicron-dominant period.

**Implications of all the available evidence**

Two doses of vaccination with BNT162b2 among adolescents are insufficient to sustain protection against symptomatic disease; however, they do offer substantial protection against serious COVID-19 outcomes for at least 3 months. Our findings support the importance of maximising vaccination coverage and the consideration of booster doses for adolescents, though further research is needed.

Studies have shown that children and adolescents are at lower risk of COVID-19-related complications than older age groups.^10^ However, high vaccination coverage in children and adolescents could potentially help avoid school absences or learning disruptions and could also protect against the long-term effects of SARS-CoV-2 infection.

Omicron replaced delta as the dominant variant in December, 2021, in Scotland and in January, 2022, in Brazil. The BNT162b2 vaccine started to be offered to adolescents in August, 2021, in Scotland and in September, 2021, in Brazil (appendix 2 pp 3–6). Brazil and Scotland differ in population size, climate, disease seasonality, and COVID-19 mitigation policy. However, both countries have high-quality SARS-CoV-2 vaccination and infection data available, giving us a unique opportunity to compare data between two populations in different contexts that result in different confounding structures, to test the hypothesis that vaccine effectiveness wanes over time among adolescents. This study assessed the associations between time since the second vaccination dose of BNT162b2 and the occurrence of symptomatic SARS-CoV-2 infection and severe COVID-19 among adolescents in Brazil and Scotland.

## Methods

### Study design

In this test-negative case-control study^11^ we estimated the vaccine effectiveness of BNT162b2 against symptomatic COVID-19 in Scotland and Brazil, and against severe COVID-19 in Brazil, by comparing the length of time since the first or second vaccine dose in individuals with a positive SARS-CoV-2 RT-PCR or antigen test (cases) and individuals with a negative SARS-CoV-2 RT-PCR or antigen test (controls). The study population were adolescents aged 12–17 years with symptoms indicative of COVID-19. In Brazil, symptoms were collected by the assisting health-care provider using the national online COVID-19 case reporting system (e-SUS Notifica), and in Scotland symptoms were self-reported and filled in on a standard online form by a health-care provider.

The date of test collection was used to stratify participants to the delta-dominant period or the omicron-dominant period. Variants were considered to be dominant when they accounted for more than 90% of sequenced viruses in Brazil and more than 50% in Scotland.^12^, ^13^ In Brazil, we included all tests from Sept 2, 2021, to April 19, 2022, and in Scotland from August 6, 2021, to April 19, 2022. In Brazil, dates from Sept 2, 2021, to Dec 31, 2021, were considered the delta-dominant period and dates from Jan 1, 2022, to April 19, 2022, were considered the omicron-dominant period. In Scotland, dates from Aug 6, 2021, to Dec 20, 2021, were considered the delta-dominant period, and dates from Dec 21, 2021, to April 19, 2022, were considered the omicron-dominant period.

In both countries, we excluded tests from individuals aged 18 years or older or younger than 11 years, asymptomatic individuals, individuals who were vaccinated before the start of the national vaccination programme, individuals who received vaccines other than BNT162b2 or a SARS-CoV-2 booster dose of any kind, and individuals for whom the time interval between the first and second dose was fewer than 21 days. Also, we excluded tests with a collection date before, or more than 10 days after, the date of the first symptom; negative SARS-CoV-2 tests within 14 days of a previous negative test; negative tests within 7 days of a positive test; any test done within 90 days after a positive test; and tests with missing sex and location information. Individuals could have more than one test, and if an adolescent presented multiple negative tests in our sample, only three negative tests from the same individual were randomly selected for inclusion using simple randomisation. Tests occurring 69 days or more after second dose were excluded. In Scotland, non-community tests (ie, tests taken in UK National Health Service [NHS] hospitals) were excluded.

We followed the RECORD reporting guidelines (appendix 2 pp 7–11),^14^ and all methods were in accordance with the Declaration of Helsinki. The statistical analysis plan is available online.^15^ For Brazil, the Brazilian National Commission in Research Ethics approved the research protocol (CONEP approval number 4.921.308 and Certificate of Presentation for Ethical Consideration registration number 50199321.9.0000.0040). CONEP waived the requirement for informed consent because we did not have access to identifiable data. The Brazilian Ministry of Health authorised the use of these data by the Vaccination Digital Vigilance (VigiVac) programme under the data protection law, which allows such use for public health research. For Scotland, ethical approval was obtained from the National Research Ethics Service Committee, Southeast Scotland 2 (12/SS/0201), and Public Benefit and Privacy Panel for Health and Social Care (PBPP; 1920–0279). The PBPP approval waived the requirement for patient consent.

### Data sources

In Brazil, data were obtained from three routinely updated sources: the national surveillance system for RT-PCR and antigen tests for COVID-19 infection (e-SUS Notifica); the information system for severe acute respiratory illness (SIVEP-Gripe), in which all COVID-19 hospital admissions and deaths are registered; and the national immunisation system (SI-PNI; appendix 2 pp 16–17). The Brazilian Ministry of Health are the sole provider of COVID-19 vaccines in Brazil and it is mandatory for private and public health providers to report COVID-19 suspected cases and hospitalisations; therefore, all Brazilians attending a health-care system should be registered in our database. We deterministically linked the data using the information provided by DATASUS from the Brazilian Ministry of Health (furthers details about linkage procedures are available in the statistical analysis plan).

For Scotland, we used data from the EAVE II platform, which holds nationwide data from 5·4 million people (approximately 99% of the national population).^16^ In the platform, primary care data are linked to laboratory and vaccination data using unique identifiers.^17^, ^18^ Clinical data collected in primary care in Scotland have consistently been of high quality (90% completeness and accuracy), and their value for epidemiological research has been repeatedly shown.^19^, ^20^, ^21^

### Exposure and confounders

Our exposure was vaccination status at the time of a negative or positive test, which was categorised as unvaccinated and, for vaccinated adolescents, grouped into periods of 0–6, 7–13, or 14 days or more after the first dose or 0–13, 14–27, 28–41, 42–55, 56–69, 70–83, 84–97, or 98 days or more after the second dose. The following confounders were included in the model for both countries: age, sex, epidemiological week, state of residence (for Brazil) or Urban Rural Classification^22^ (for Scotland), socioeconomic position measured by quintile of deprivation (using the Índice Brasileiro de Privação^23^ in Brazil and the Scottish Index of Multiple Deprivation in Scotland, which analyse and monitor health inequalities by location^24^), previous SARS-CoV-2 infection (either none, between 3 and 6 months previously, or more than 6 months previously), and number of comorbidities commonly associated with COVID-19 illness (appendix 2 p 17). In Brazil, we also included current pregnancy or being in the post-partum period (ie, up to 45 days after giving birth) and ethnicity as potential confounders.

### Outcomes

The primary outcome was COVID-19 symptomatic infection, confirmed by rapid antigen testing or RT-PCR in Brazil and only by RT-PCR in Scotland. Additionally, we evaluated severe COVID-19 (hospital admission or death), defined as a positive test that occurred within 14 days before and up to 3 days after the date of hospital admission or death occurring within 28 days of a positive test. The severe COVID-19 analysis was restricted to Brazil due to the small number of severe COVID-19-related cases in Scotland.

### Statistical analysis

The odds ratio (OR) comparing the odds of vaccination between cases and controls and its associated 95% CI were derived using logistic regression. Vaccine effectiveness was estimated as (1–OR) × 100, obtained from an adjusted model including the described covariates, expressed as a percentage. The estimates for each covariate used in the adjusted model are presented in appendix 2 (pp 12–14). All data processing and analyses were done using R (version 4.1.1) using the Tidyverse package.^25^ The models were fitted separately to each variant period, including only the tests that were done during the dominant period. In Brazil, missing values related to ethnicity were imputed using multiple imputation as sensitivity analyses. For these analyses, we used the MICE package (version 1.16) with five imputations.^26^

### Role of the funding source

The funder of the study had no role in study design, data collection, data analysis, data interpretation, or writing of the report.

## Results

**Figure 1:**
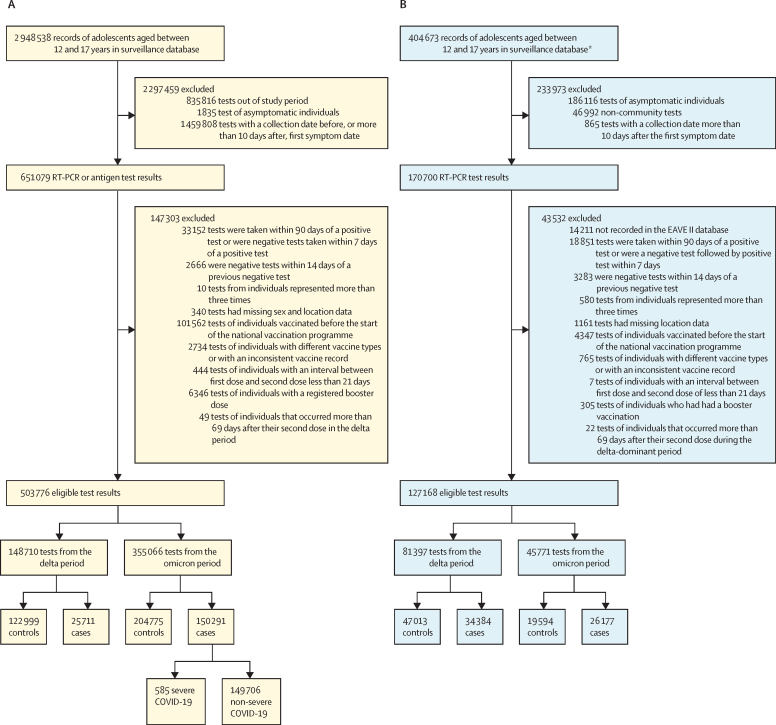
Flow chart to select cases and controls for in Brazil (A) and Scotland (B) *Excluding 234 886 tests taken outside of the study period.

We analysed 503 776 eligible SARS-CoV-2 tests of 2 948 538 symptomatic adolescents in Brazil taken from Sept 2, 2021, to April 19, 2022. Of the tests, there were 176 002 (34·9%) positive tests and 327  774 (65·1%) negative tests (figure 1A). 355 066 tests were recorded during the omicron period, in which 150 291 (42·3%) were cases and 204 775 (57·7%) were controls. Of these 150 291 omicron-period positive tests, 585 (0·4%) were of individuals with severe COVID-19. We analysed 127 168 eligible tests taken by 404  673 adolescents in Scotland from August 6, 2021, to April 19, 2022 (figure 1B). Of these tests, 60 574 (47·6%) were positive and 66 616 (52·4%) were negative. 45 771 tests were recorded during the omicron period in Scotland, of which 26 177 (57·2%) were cases and 19 594 (42·8%) were controls. The distribution according to age, sex, socioeconomic position, comorbidities, and hospital admission was similar between the adolescents who tested positive and negative in both countries during the omicron period (table 1; appendix 2 p 15) and the delta period (appendix 2 p 15).

**Table 1:** Participant characteristics during the omicron-dominant period in Brazil and Scotland

	**Brazil**		**Scotland**	
	Cases (n=150 291)	Controls (n=204 775)	Cases (n=26 177)	Controls (n=19 594)
**Age, years**				
12	22 623 (15·1%)	30 263 (14·8%)	4539 (17·3%)	3441 (17·6%)
13	20 936 (13·9%)	31 189 (15·2%)	4277 (16·3%)	3626 (18·5%)
14	22 838 (15·2%)	31 949 (15·6%)	4287 (16·4%)	3445 (17·6%)
15	25 614 (17·0%)	35 606 (17·4%)	4184 (16·0%)	3199 (16·3%)
16	28 402 (18·9%)	37 636 (18·4%)	4507 (17·2%)	3156 (16·1%)
17	29 878 (19·9%)	38 132 (18·6%)	4383 (16·7%)	2727 (13·9%)
**Sex**				
Female	82 880 (55·1%)	109 217 (53·3%)	14 271 (54·5%)	10 423 (53·2%)
Male	67 411 (44·9%)	95 558 (46·7%)	11 906 (45·5%)	9171 (46·8%)
Deprivation index, quintile*				
1	45 618 (30·4%)	74 089 (36·2%)	6261 (23·9%)	3986 (20·3%)
2	27 546 (18·3%)	37 674 (18·4%)	5497 (21·0%)	3818 (19·5%)
3	30 011 (20·0%)	36 818 (18·0%)	4586 (17·5%)	3494 (17·8%)
4	28 759 (19·1%)	31 295 (15·3%)	4928 (18·8%)	4091 (20·9%)
5	18 357 (12·2%)	24 899 (12·2%)	4905 (18·7%)	4205 (21·5%)
**Number of comorbidities**				
0	146 174 (97·3%)	197 309 (96·4%)	21 350 (81·6%)	15 568 (79·5%)
1	3973 (2·6%)	7275 (3·6%)	4390 (16·8%)	3610 (18·4%)
≥2	144 (0·1%)	191 (0·1%)	437 (1·7%)	416 (2·1%)
**Previous confirmed infection**				
No	146 843 (97·7%)	195 295 (95·4%)	24 943 (95·3%)	17 783 (90·8%)
Yes, in the previous 3–6 months	460 (0·3%)	1254 (0·6%)	129 (0·5%)	166 (0·8%)
Yes, more than 6 months previous	2988 (2·0%)	8226 (4·0%)	1105 (4·2%)	1645 (8·4%)
**Admitted to hospital**				
No	149 731 (99·6%)	203 980 (99·6%)	26 077 (99·6%)	19 544 (99·7%)
Yes	560 (0·4%)	795 (0·4%)	100 (0·4%)	50 (0·3%)
**Died**				
No	150 237 (>99·9%)	204 725 (>99·9%)	26 177 (100%)	19 594 (100%)
Yes	54 (<0·1%)	50 (<0·1%)	0	0

* Deprivation was measured using the Índice Brasileiro de Privação^21^ in Brazil and the Scottish Index of Multiple Deprivation in Scotland.^22^

**Figure 2:**
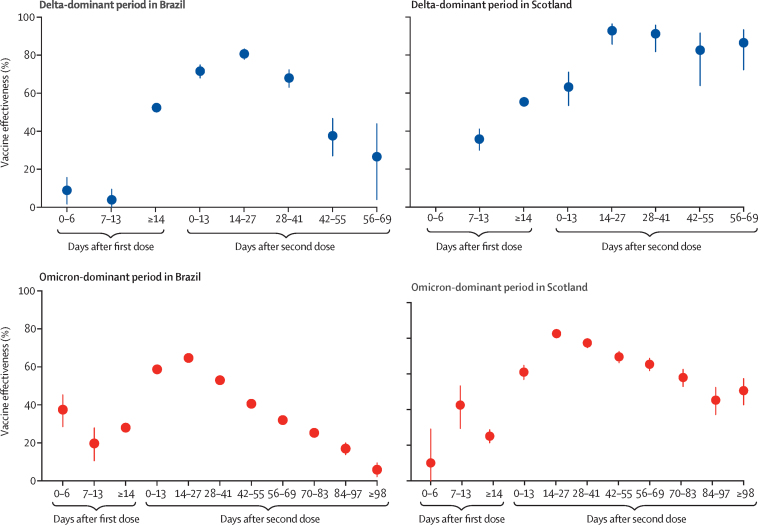
Vaccine effectiveness against symptomatic infection by time since the first and second doses of BNT162b2 during the delta-dominant and omicron-dominant periods in Brazil and Scotland Bars indicate 95% CIs

During the delta-dominant period in Brazil, vaccine effectiveness for symptomatic infection in adolescents after two doses of BNT162b2 peaked at 14–27 days (80·7% [95% CI 77·8–83·3]; table 2; figure 2). However, protection started to decline after 27 days (at 28–41 days protection was 68·0% [63·2–72·3]), reducing to 26·6% (4·1–43·9) at 56–69 days. In Scotland, protection for symptomatic infection in the delta-dominant period was also highest at 14–27 days after the second dose (92·8% [95% CI 85·7–96·4]) and reduced at 56–69 days (86·5%; 95% CI 72·2–93·4), although the reduction in effectiveness at 56–69 days compared with at 14–27 days was less dramatic in Scotland than in Brazil (table 2; figure 2).

**Table 2:** ORs and BNT162b2 vaccine effectiveness against symptomatic infection during the delta-dominant period in Brazil and Scotland

		**Brazil**					**Scotland**				
		Positive tests (n=25 711)	Negative tests (n=122 999)	Crude OR (95% CI)	Adjusted OR (95% CI)	Vaccine effectiveness (%; 95% CI)	Positive tests (n=34 384)	Negative tests (n=47 013)	Crude OR (95% CI)	Adjusted OR (95% CI)	Vaccine effectiveness (%; 95% CI)
Number of tests from unvaccinated individuals		15 544/59 268 (26·2%)	43 724/59 268 (73·8%)	..	..	..	25 717/54 993 (46·8%)	29 276/54 993 (53·2%)	..	..	..
Time after first dose											
	0–6 days	970/4225 (23·0%)	3255/4225 (77·0%)	0·80 (0·74 to 0·86)	0·91 (0·84 to 0·98)	8·9 (1·7 to 15·7)	1254/2487 (50·4%)	1233/2487 (49·6%)	1·07 (0·99 to 1·17)	1·07 (0·98 to 1·16)	−6·9 (−16·3 to 1·8)
	7–13 days	1725/7399 (23·3%)	5674/7399 (76·7%)	0·84 (0·80 to 0·89)	0·96 (0·91 to 1·02)	3·9 (−2·0 to 9·5)	967/2479 (39·0%)	1512/2479 (61·0%)	0·67 (0·61 to 0·73)	0·64 (0·59 to 0·70)	35·8 (30·0 to 41·1)
	≥14 days	6392/61 170 (10·4%)	54 778/61 170 (89·6%)	0·39 (0·37 to 0·40)	0·48 (0·46 to 0·50)	52·4 (50·5 to 54·3)	6297/20 764 (30·3%)	14 467/20 764 (69·7%)	0·47 (0·45 to 0·49)	0·45 (0·43 to 0·47)	55·4 (53·4 to 57·3)
Time after second dose											
	0–13 days	317/5663 (5·6%)	5346/5663 (94·4%)	0·22 (0·20 to 0·25)	0·28 (0·25 to 0·32)	71·6 (68·0 to 74·9)	114/354 (32·2%)	240/354 (67·8%)	0·38 (0·30 to 0·48)	0·37 (0·29 to 0·47)	63·2 (53·4 to 71·0)
	14–27 days	227/5463 (4·2%)	5236/5463 (95·8%)	0·16 (0·14 to 0·18)	0·19 (0·17 to 0·22)	80·7 (77·8 to 83·3)	9/116 (7·8%)	107/116 (92·2%)	0·08 (0·04 to 0·15)	0·07 (0·04 to 0·14)	92·8 (85·7 to 96·4)
	28–41 days	248/3368 (7·4%)	3120/3368 (92·6%)	0·26 (0·23 to 0·30)	0·32 (0·28 to 0·37)	68·0 (63·2 to 72·3)	8/92 (8·7%)	84/92 (91·3%)	0·09 (0·05 to 0·20)	0·09 (0·04 to 0·18)	91·2 (81·8 to 95·8)
	42–55 days	214/1734 (12·3%)	1520/1734 (87·7%)	0·44 (0·38 to 0·52)	0·62 (0·53 to 0·73)	37·6 (27·0 to 46·7)	9/52 (17·3%)	43/52 (82·7%)	0·19 (0·09 to 0·39)	0·17 (0·08 to 0·36)	82·6 (63·9 to 91·6)
	56–69 days	74/420 (17·6%)	346/420 (82·4%)	0·57 (0·44 to 0·74)	0·73 (0·56 to 0·96)	26·6 (4·1 to 43·9)	9/60 (15·0%)	51/60 (85·0%)	0·15 (0·08 to 0·31)	0·14 (0·07 to 0·28)	86·5 (72·2 to 93·4)

The delta-dominant period in Brazil was from Sept 2, 2021, to Dec 31, 2021, and in Scotland the delta-dominant period was from Aug 6, 2021, to Dec 20, 2021. OR=odds ratio. 95% CIs could not be estimated in tests occurring more than 69 days after the second dose, due to small number of events in cases and controls.

Vaccines were less effective against the omicron variant than the delta variant at all intervals after two-dose vaccination in both countries. During the omicron-dominant period, after the second BNT162b2 dose, vaccine effectiveness peaked between 14–27 days in Brazil (64·7% [95% CI 63·0–66·3]; table 3; figure 2). Protection started to decline after 27 days (at 28–41 days protection was 53·0% [51·3–54·7]), reducing to 5·9% (95% CI 2·2–9·4) at 98 days and longer. In Scotland, vaccine effectiveness for symptomatic infection during the omicron-dominant period also peaked at 14–27 days after the second dose (82·6% [95% CI 80·6–84·5]), and after 98 days and more was 50·6% (42·7–57·4; table 3; figure 2).

**Table 3:** ORs and BNT162b2 vaccine effectiveness against symptomatic infection during the omicron-dominant period in Brazil and Scotland

		**Brazil**					**Scotland**				
		Positive tests (n=150 291)	Negative tests (n=204 775)	Crude OR (95% CI)	Adjusted OR (95% CI)	Vaccine effectiveness (%; 95% CI)	Positive tests (n=26 177)	Negative tests (n=19 594)	Crude OR (95% CI)	Adjusted OR (95% CI)	Vaccine effectiveness (%; 95% CI)
Number of tests from unvaccinated individuals		24 933/47 903 (52·0%)	22 970/47 903 (48·0%)	..	..	..	9522/14 377 (6·2%)	4855/14 377 (33·8%)	..	..	..
Time after first dose											
	0–6 days	435/955 (45·5%)	520/955 (54·5%)	0·59 (0·52 to 0·68)	0·63 (0·55 to 0·71)	37·5 (28·5 to 45·3)	210/325 (64·6%)	115/325 (35·4%)	0·88 (0·70 to 1·11)	0·90 (0·71 to 1·14)	10·1 (−14·2 to 29·1)
	7–13 days	776/1494 (51·9%)	718/1494 (48·1%)	0·80 (0·72 to 0·89)	0·80 (0·72 to 0·89)	19·7 (10·6 to 27·9)	219/404 (54·2%)	185/404 (45·8%)	0·55 (0·45 to 0·68)	0·57 (0·47 to 0·71)	42·5 (29·4 to 53·3)
	≥14 days	42 488/98 628 (43·1%)	56 140/98 628 (56·9%)	0·71 (0·70 to 0·73)	0·72 (0·70 to 0·74)	28·0 (26·3 to 29·7)	10 904/17 505 (62·3%)	6601/17 505 (37·7%)	0·78 (0·74 to 0·82)	0·75 (0·71 to 0·79)	25·1 (21·3 to 28·7)
Time after second dose											
	0–13 days	2410/6772 (35·6%)	4362/6772 (64·4%)	0·42 (0·40 to 0·44)	0·41 (0·39 to 0·44)	58·7 (56·4 to 61·0)	891/1970 (45·2%)	1079/1970 (54·8%)	0·41 (0·37 to 0·45)	0·39 (0·35 to 0·43)	61·0 (56·9 to 64·8)
	14–27 days	3469/11 169 (31·1%)	7700/11 169 (68·9%)	0·36 (0·35 to 0·38)	0·35 (0·34 to 0·37)	64·7 (63·0 to 66·3)	494/1829 (27·0%)	1335/1829 (73·0%)	0·18 (0·17 to 0·21)	0·17 (0·15 to 0·19)	82·6 (80·6 to 84·5)
	28–41 days	8060/21 486 (37·5%)	13 426/21 486 (62·5%)	0·49 (0·47 to 0·51)	0·47 (0·45 to 0·49)	53·0 (51·3 to 54·7)	540/1700 (31·8%)	1160/1700 (68·2%)	0·24 (0·21 to 0·27)	0·23 (0·20 to 0·25)	77·4 (74·7 to 79·8)
	42–55 days	15 631/34 846 (44·9%)	19 215/34 846 (55·1%)	0·61 (0·59 to 0·63)	0·59 (0·58 to 0·61)	40·6 (38·8 to 42·4)	754/1963 (38·4%)	1209/1963 (61·6%)	0·33 (0·30 to 0·37)	0·30 (0·27 to 0·34)	69·6 (66·3 to 72·6)
	56–69 days	19 627/40 463 (48·5%)	20 836/40 463 (51·5%)	0·67 (0·65 to 0·69)	0·68 (0·66 to 0·70)	32·0 (30·0 to 33·9)	915/2256 (40·6%)	1341/2256 (59·4%)	0·38 (0·34 to 0·42)	0·35 (0·31 to 0·38)	65·4 (61·9 to 68·7)
	70–83 days	15 069/31 243 (48·2%)	16 174/31 243 (51·8%)	0·75 (0·72 to 0·77)	0·75 (0·72 to 0·77)	25·3 (22·9 to 27·6)	717/1542 (46·5%)	825/1542 (53·5%)	0·47 (0·42 to 0·52)	0·42 (0·37 to 0·47)	58·0 (52·9 to 62·6)
	84–97 days	9045/21 979 (41·2%)	12 934/21 979 (58·9%)	0·81 (0·78 to 0·84)	0·83 (0·80 to 0·86)	17·0 (13·8 to 20·0)	549/1013 (54·2%)	464/1013 (45·8%)	0·60 (0·52 to 0·68)	0·55 (0·48 to 0·63)	45·3 (37·2 to 52·4)
	≥98 days	8348/38 128 (21·9%)	29 780/38 128 (78·1%)	0·91 (0·88 to 0·95)	0·94 (0·91 to 0·98)	5·9 (2·2 to 9·4)	462/887 (52·1%)	425/887 (47·9%)	0·55 (0·48 to 0·64)	0·49 (0·43 to 0·57)	50·6 (42·7 to 57·4)

The omicron-dominant period in Brazil was from Jan 1, 2022, to April 19, 2022, and in Scotland the omicron-dominant period was from Dec 21, 2021, to April 19, 2022. OR=odds ratio.

In Brazil, the estimated vaccine effectiveness against severe COVID-19 during the omicron-dominant period 14 days or longer after the first dose was 56·3% (95% CI 45·9–64·6; table 4). From 14 days to 27 days after the second dose, protection increased to 75·6% (58·1–85·8). More than 27 days after the second dose, vaccine effectiveness reached more than 80% and remained at a similar level of protection at 98 days and longer after the second dose (82·7% [68·8–90·4]; appendix 2 p 16). In Brazil, the ethnicity register is optional, and there are 93 231 (18·5%) of 503 776 missing data in this category. The sensitivity analyses based on multiple imputations produced similar results to the primary analyses (appendix 2 p 16).

**Table 4:** ORs and BNT162b2 vaccine effectiveness against severe COVID-19 during the omicron-dominant period in Brazil

		**Patients with severe COVID-19 (n=585)**	**Negative tests (N=204 775)**	**Crude OR (95% CI)**	**Adjusted OR (95% CI)**	**Vaccine effectiveness (%; 95% CI)**
Number of tests from unvaccinated individuals		205/23 175 (0·9%)	22 970/23 175 (99·1%)	..	..	..
Time after first dose						
	0–6 days	4/524 (0·8%)	520/524 (99·2%)	0·76 (0·28 to 2·05)	0·80 (0·25 to 2·53)	20·6 (−152·2 to 75·0)
	7–13 days	3/721 (0·4%)	718/721 (99·6%)	0·43 (0·14 to 1·34)	0·38 (0·12 to 1·23)	62·4 (−22·2 to 88·5)
	≥14 days	208/56 348 (0·4%)	56 140/56 348 (99·6%)	0·37 (0·31 to 0·45)	0·44 (0·35 to 0·54)	56·3 (45·9 to 64·6)
Time after second dose						
	0–13 days	13/4375 (0·3%)	4362/4375 (99·7%)	0·25 (0·14 to 0·44)	0·35 (0·20 to 0·63)	65·0 (37·2 to 80·5)
	14–27 days	15/7715 (0·2%)	7700/7715 (99·8%)	0·18 (0·10 to 0·30)	0·24 (0·14 to 0·42)	75·6 (58·1 to 85·8)
	28–41 days	20/13 446 (0·1%)	13 426/13 446 (99·9%)	0·13 (0·08 to 0·21)	0·17 (0·11 to 0·28)	82·8 (72·1 to 89·4)
	42–55 days	30/19 245 (0·2%)	19 215/19 245 (99·8%)	0·12 (0·08 to 0·18)	0·16 (0·11 to 0·24)	84·2 (76·3 to 89·5)
	56–69 days	34/20 870 (0·2%)	20 836/20 870 (99·8%)	0·12 (0·08 to 0·17)	0·16 (0·11 to 0·24)	83·7 (76·0 to 88·9)
	70–83 days	27/16 201 (0·2%)	16 174/16 201 (99·8%)	0·13 (0·09 to 0·20)	0·18 (0·12 to 0·27)	82·0 (72·6 to 88·2)
	84–97 days	12/12 946 (0·1%)	12 934/12 946 (99·9%)	0·11 (0·06 to 0·19)	0·14 (0·07 to 0·25)	86·4 (75·2 to 92·6)
	≥98 days	14/29 794 (<0·1%)	29 780/29 794 (>99·9%)	0·13 (0·07 to 0·24)	0·17 (0·10 to 0·31)	82·7 (68·8 to 90·4)

The omicron-dominant period in Brazil was from Jan 1, 2022, to April 19, 2022. OR=odds ratio.

## Discussion

Our analyses of Brazilian and Scottish national data showed that BNT162b2 vaccine effectiveness against symptomatic COVID-19 in adolescents aged 12–17 years declined over time since the second dose and varied by the predominant circulating SARS-CoV-2 variant (the vaccine effectiveness was substantially lower during the omicron-dominant period than the delta-predominant period). Vaccine effectiveness against symptomatic SARS-CoV-2 infection decreased to 5·9% in Brazil and 50·6% in Scotland at 98 days or more after the second dose during the omicron-dominant period. In Brazil, protection against severe disease after two doses of vaccine was well maintained, remaining above 80% at 98 days or more after the second dose. Unfortunately, we could not determine protection against severe forms of the disease in Scotland due to the small number of individuals with severe COVID-19 in Scotland during the study.

Neutralising antibody levels are highly predictive of immune protection from symptomatic SARS-CoV-2 infection.^27^ Previous studies in adults have shown a significant reduction in neutralising activity against omicron compared with earlier pandemic variants in serum of individuals who have received two doses of BNT162b2.^28^, ^29^ As antibodies wane over time since vaccination, the risk of infection might increase. However, cellular immune responses probably have a role in protecting and preventing the progression to severe disease.^30^ These conclusions are consistent with the findings from this study of adolescents; although vaccine effectiveness against symptomatic infection decreased, the protection for severe outcomes was sustained.

Previous studies have reported waning of vaccine effectiveness against symptomatic SARS-CoV-2 infection in adolescents. Data from England on vaccine effectiveness over time against symptomatic infection among people aged 16–17 years showed that after dose two, vaccine effectiveness during the delta-dominant period peaked between 14 and 34 days at 96·1% (95% CI 95·2–96·8) and during the omicron-dominant period between 7 and 13 days following the second dose at 76·1% (73·4–78·6). However, effectiveness during the omicron-dominant period reduced rapidly, reaching 22·6% (95% CI 14·5–29·9) at day 70 and onwards compared with 83·7% (72·0–90·5) during the delta-dominant period at day 70 and onwards.^31^ For adolescents aged 12–15 years, vaccine effectiveness during the delta-dominant period at 14 days or more after two doses was 87·2% and during the omicron-dominant period was 73·0%; no further follow-up was available.^32^ In the USA, adolescents aged 12–17 years had no significant protection against infection at 150 days or more after two doses during the omicron-dominant period.^9^ Another US study observed that, among adolescents aged 12–15 years, vaccine effectiveness was 59·0% (95% CI 22·0–79·0) at 14–149 days after the second dose during the omicron-dominant period.^32^

Data on vaccine effectiveness against severe disease over time among adolescents are scarce. In the study done in England, vaccine effectiveness against hospitalisation at 28 days after the first dose was 76·3% (95% CI 61·1–85·6) for those aged 16–17 years and 83·4% (54·0–94·0) for those aged 12–15 years during the delta-dominant period.^31^ Longer follow-up or data during the omicron-dominant period were not available. In our study, similarly high protection was observed against severe COVID-19 (hospital admission and death) during the omicron-dominant period, and this protection was sustained over time. The present report on outcomes in Brazil is the first nationwide study to evaluate the duration of vaccine effectiveness against severe COVID-19 in adolescents during the omicron-dominant period to date.

Cross-country comparison studies have important strengths. Drawing on data from Brazil and Scotland means results are less likely to be explained by confounding, because the timing of waves of infection and the timing of vaccination differ across countries. Additional strengths include the use of high-quality national databases, which increases statistical power. By using a test-negative design, we have minimised bias related to access to health care, the occurrence of symptoms, and health-seeking behaviour.

However, our study has several limitations. A limitation intrinsic to the use and availability of secondary data is the restricted choice of covariates and the potential for misclassifying vaccine status due to linkage failure. Our estimates of vaccine effectiveness against severe COVID-19 are subject to considerable uncertainty due to the relatively small number of outcome events; therefore, these estimates should be interpreted with caution. In this analysis, our reference group was unvaccinated people; the characteristics of this group probably differ from those of vaccinated adolescents, which could confound our estimates of vaccine effectiveness. Misclassification of testing results and variant sequencing might also have occurred, which could explain the accentuated protection declining during the delta-dominant period in Brazil. Vaccine effectiveness estimated in Brazil was lower than the corresponding vaccine effectiveness in Scotland, which is probably explained by the increased background risk of COVID-19 transmission in Brazil, which is the result of many factors such as public health interventions.^8^, ^33^ Due to the high proportion of asymptomatic infections among children and adolescents, and testing constraints in Brazil, it would be challenging to estimate the role of hybrid immunity.^34^ Our vaccine effectiveness estimates during the delta-dominant period included time periods in which not all members of the population were eligible for all exposure levels. Therefore, during the delta-dominant period, the positivity assumption was not met,^35^ and estimates were necessarily based on extrapolations. These estimates could therefore have potentially been subject to bias. Although our analysis adjusts for previous infection, we did not study the effect of heterogeneity in vaccine effectiveness across population subgroups, which could potentially be explored using alternative study designs.^36^ Furthermore, despite our model adjustment by state (Brazil) and Urban Rural Classification (Scotland) there could still be regional heterogeneity in infections rates. Our model is limited to a short time series and, therefore, does not account for seasonality. Lastly, in the Brazilian statistical analysis plan, we also aimed to estimate vaccine effectiveness for CoronaVac (Sinovac); however, because of lower rates of this vaccine administered in this population during the study period, we did not have enough data and so restricted the analyses to BNT162b2.

In summary, our findings indicate that protection against symptomatic infection with the omicron variant rapidly decreases over time after two doses of BNT162b2 in adolescents, and therefore, two doses are insufficient to sustain protection against symptomatic disease. However, protection against severe disease probably remains high at 98 days or more after the second dose. Our findings support the importance of maximising vaccination coverage and for considering booster doses for adolescents. Further studies will be needed to assess the duration of protection and the need for booster doses.

## Data sharing

Our agreement with the Brazilian Ministry of Health for accessing the referenced databases patently denies authorisation of access to any third parties. All requests to access these databases must be addressed to the Ministry of Health. Unlinked data are available at https://opendatasus.saude.gov.br. Data dictionaries, linkage scripts, and analyses scripts are available at https://vigivac.fiocruz.br and https://github.com/cidacslab/vigivac. Regarding Scottish data, a data dictionary covering the datasets used in this study can be found at https://github.com/EAVE-II/EAVE-II-data-dictionary. Patient-level data underlying these data are controlled and cannot be shared publicly due to data protection and confidentiality requirements. Data can be made available to approved researchers for analysis after securing relevant permissions from the data holders via the Public Benefit and Privacy Panel. Enquiries regarding data availability should be directed to phs.edris@phs.scot. All code used on the Scottish data in this study is publicly available at https://github.com/EAVE-II.

## Declaration of interests

MB-N reports grants from the Fazer o Bem Faz Bem programme from JBS SA. VdAO, VSB, MLB, and MB-N are employees of Fiocruz, a federal public institution that manufactures Vaxzevria in Brazil through a full technology transfer agreement with AstraZeneca. Fiocruz allocates all its manufactured products to the Ministry of Health for public health use. SVK was Co-Chair of the Scottish Government's Expert Reference Group on Ethnicity and COVID-19 and a member of the UK Government's Scientific Advisory Group on Emergencies subgroup on ethnicity. IR is a member of the Scientific Advisory Committee of the Government of Croatia and co-Editor-in-Chief of the *Journal of Global Health*. CRS declares funding from the Medical Research Council, the National Institute for Health Research, the Chief Scientist Office, and the New Zealand Ministry for Business, Innovation and Employment and Health Research Council during the conduct of this study. CR declares he is a Member of SPI-M, Scottish Government Scientific Advisory Committee, MHRA COVID-19 vaccine benefit and risk expert working group. AS declares that he is a member of the UK and Scottish Governments COVID-19 Advisory Boards and Astra-Zeneca's Thrombotic Thrombocytopenic Taskforce. IR is Co-Editor-in-Chief of the *Journal of Global Health* and President of the International Society of Global Health. All other authors declare no competing interests.

## Supplementary Materials

Portuguese translation of the abstract
