# Supplementary material for: Vaccine effectiveness of two-dose BNT162b2 against symptomatic and severe COVID-19 among adolescents in Brazil and Scotland over time: a test-negative case-control study
Source: Lancet Infect Dis. 2022 Nov;22(11):1577–86. doi: 10.1016/S1473-3099(22)00451-0 (PMC9359673; doi:10.1016/S1473-3099(22)00451-0)
Supplement: Portuguese translation of the abstract [file mmc1.pdf]

THE LANCET
Infectious Diseases

Supplementary appendix 1

This translation in Portuguese was submitted by the authors and we reproduce it as supplied. It has not been peer reviewed. *The Lancet*'s editorial processes have only been applied to the original in English, which should serve as reference for this manuscript.

Esta tradução em português foi submetida pelos autores e nós não fizemos quaisquer alterações. Esta versão não foi revista por pares. O processo editorial do *The Lancet* só foi aplicado à versão original em inglês, que deve servir como referência para este artigo.

Supplement to: Florentino PTV, Millington T, Cerqueira-Silva T, et al. Vaccine effectiveness of two-dose BNT162b2 against symptomatic and severe COVID-19 among adolescents in Brazil and Scotland over time: a test-negative case-control study. *Lancet Infect Dis* 2022; published online Aug 8. https://doi.org/10.1016/S1473-3099(22)00451-0.

Resumo

Contexto

Pouco se sabe sobre a efetividade da vacina ao longo do tempo entre os adolescentes, especialmente contra a variante SARS-CoV-2 Ômicron (B.1.1.529). Este estudo avaliou as associações entre o tempo desde a vacinação de duas doses com BNT162b2 e a ocorrência de infecção sintomática por SARS-CoV-2 e formas graves de COVID-19 em adolescentes no Brasil e na Escócia.

Métodos

Fizemos um estudo teste negativo caso-controle em adolescentes de 12 a 17 anos com sintomas relacionados ao COVID-19 no Brasil e na Escócia. Vinculamos registros de testes SARS-CoV-2 RT-PCR e de antígenos a registros nacionais de vacinação e registros clínicos. Excluímos testes de indivíduos que não apresentavam sintomas, foram vacinados antes do início do programa nacional de vacinação, receberam vacinas diferentes de BNT162b2 ou uma dose de reforço de SARS-CoV-2 de qualquer tipo, ou tiveram um intervalo entre a primeira e a segunda dose inferior a 21 dias. Além disso, excluímos testes negativos de SARS-CoV-2 registrados em 14 dias após um teste negativo anterior, testes negativos registrados em 7 dias após um teste positivo, qualquer teste realizado dentro de 90 dias após um teste positivo e testes sem registros de sexo e localização. Casos (adolescentes com teste positivo para SARS-CoV-2) e controles (adolescentes com teste negativo) foram selecionados de uma amostra de indivíduos nos quais os testes foram coletados dentro de 10 dias do início dos sintomas. Estimamos a razão de probabilidade ajustada e a efetividade da vacina contra COVID-19 sintomática para ambos os países e contra formas graves de COVID-19 (hospitalização ou morte) para o Brasil em períodos quinzenais.

Resultados

Analisamos 503.776 testes de 2.948.538 adolescentes no Brasil entre 2 de setembro de 2021 e 19 de abril de 2022 e 127.190 testes de 404.673 adolescentes na Escócia entre 6 de agosto de 2021 e 19 de abril de 2022. A efetividade da vacina atingiu o pico de 14 – 27 dias após a segunda dose em ambos os países durante ambas as ondas, e foi significativamente menor contra infecção sintomática durante o período Ômícron dominante no Brasil (64,7% [IC 95% 63,0–66,3]) e na Escócia (82,6% [80,6–84,5]), do que no período delta-dominante (80,7% [IC 95% 77,8–83,3] no Brasil e 92,8% [85·7–96·4] na Escócia). A efetividade da vacina começou a diminuir a partir de 27 dias após a segunda dose para ambos os países, reduzindo para 5,9% (IC 95% 2·2–9·4) no Brasil e

50,6% (42·7–57·4) na Escócia em 98 dias ou mais durante o período de Ômícron dominante. No Brasil, a proteção contra doença grave permaneceu acima de 80% a partir de 28 dias após a segunda dose e foi de 82,7% (IC 95% 68,8–90,4) em 98 dias ou mais após receber a segunda dose.

Interpretação

Encontramos diminuição da proteção vacinal de BNT162b2 contra infecção sintomática por COVID-19 entre adolescentes no Brasil e na Escócia a partir de 27 dias após a segunda dose. No entanto, a proteção contra formas graves de COVID-19 permaneceu alta em 98 dias ou mais após a segunda dose no período dominante de Ômícron. Doses de reforço para adolescentes precisam ser consideradas.

Financiamento

UK Research and Innovation (Medical Research Council), Scottish Government, Health Data Research UK BREATHE Hub, Fiocruz, Fazer o Bem Faz Bem programme, Brazilian National Research Council, and Wellcome Trust.
