# Supplementary material for: Vaccine effectiveness of two-dose BNT162b2 against symptomatic and severe COVID-19 among adolescents in Brazil and Scotland over time: a test-negative case-control study
Source: Lancet Infect Dis. 2022 Nov;22(11):1577–86. doi: 10.1016/S1473-3099(22)00451-0 (PMC9359673; doi:10.1016/S1473-3099(22)00451-0)
Supplement: Supplementary appendix 2 [file mmc2.pdf]

THE LANCET
Infectious Diseases

Supplementary appendix 2

This appendix formed part of the original submission and has been peer reviewed. We post it as supplied by the authors.

Supplement to: Florentino PTV, Millington T, Cerqueira-Silva T, et al. Vaccine effectiveness of two-dose BNT162b2 against symptomatic and severe COVID-19 among adolescents in Brazil and Scotland over time: a test-negative case-control study. *Lancet Infect Dis* 2022; published online Aug 8. https://doi.org/10.1016/S1473-3099(22)00451-0.

Supplementary appendix to:

Vaccine effectiveness of two-dose BNT162b2 over time against COVID-19 symptomatic infection and severe cases among adolescents: Test negative design case control studies in Brazil and Scotland

Pilar T V Florentino PhD*[1,2], Tristan Millington PhD*[3], Thiago Cerqueira-Silva MD[4,5], Prof. Chris Robertson PhD[6], Vinicius de Araújo Oliveira MD[1], Juracy B S Júnior MSc[7], Flávia J O Alves PhD[1], Gerson O Penna PhD[8], Prof. Srinivasa Vital Katikireddi PhD[9], Prof. Viviane S Boaventura PhD[4,5], Prof. Guilherme L Werneck DSc[11,12], Prof. Neil Pearce PhD[13], Colin McCowan PhD[14], Christopher Sullivan PhD[6], Utkarsh Agrawal PhD[14], Zoe Grange PhD[6], Sir Lewis D. Ritchie MD[15], Colin R Simpson PhD[16], Prof. Aziz Sheikh MD[3], Prof. Mauricio L Barreto MD[1], Prof. Igor Rudan PhD†[3], Prof. Manoel Barral-Netto PhD†[17], Enny S Paixão PhD†[13]

Affiliations
[1] Centre of Data and Knowledge Integration for Health (CIDACS), Gonçalo Moniz Institute, Oswaldo Cruz Foundation, Salvador, Brazil
[2] Biomedical Science Institute, University of São Paulo, São Paulo, Brazil
[3] Usher Institute, University of Edinburgh, Edinburgh, UK
[4] LIB and LEITV Laboratories, Instituto Gonçalo Moniz, Salvador, Brazil
[5] Federal University of Bahia, Salvador, Brazil
[6] Public Health Scotland, Glasgow, Scotland, UK
[7] Institute of Collective Health, Federal University of Bahia, Salvador, Brazil
[8] Tropical Medicine Centre, University of Brasília, Fiocruz School of Government Brasília, Brazil
[9] MRC/CSO Social & Public Health Sciences Unit, University of Glasgow, Glasgow, UK
[11] Department of Epidemiology, Social Medicine Institute, State University of Rio de Janeiro, Rio de Janeiro, Brazil.
[12] Institute of Collective health studies, Federal University of Rio de Janeiro, Rio de Janeiro, Brazil
[13] London School of Hygiene and Tropical Medicine, London, UK
[14] School of Medicine, University of St Andrews, St Andrews, UK
[15] Academic Primary Care, University of Aberdeen, Aberdeen, UK
[16] School of Health, Wellington Faculty of Health, Victoria University of Wellington, Wellington, New Zealand
[17] Gonçalo Moniz Institute, Oswaldo Cruz Foundation, Salvador, Brazil

Contents

Table S1. Vaccination plan for adolescents aged 12 to 17 years in Brazil. **3**

Table S2. Vaccination plan for adolescents aged 12 to 17 years in Scotland. **4**

Table S3. Checklist: The RECORD statement **7**

Table S4. Estimates for covariates in logistic regression model for Omicron and Delta in Brazil ... **12**

Table S5. Estimates for covariates in logistic regression model for Omicron and Delta in Scotland **13**

Supplemental table 6. Table of characteristic during the Delta period for Brazil and Scotland **15**

Table S7. Specific demographic for Brazil in Omicron and Delta Period **15**

Table S8. Vaccine effectiveness based on multiple imputations analyses. **16**

Figure S1. VE from severe cases (hospitalisation or death) by the length of time since the first and second doses of BNT162b2 in Brazil during the Omicron dominant period. **16**

Supplemental methods **16**

References **18**

Table S1. Vaccination plan for adolescents aged 12 to 17 years in Brazil.

Date	Technical notes issued by the Ministry of Health	Recommendations
10/06/2021- ANVISA Authorization for the Pfizer vaccine in adolescents aged 12 to 17 years		
02/09/2021	**NOTA TÉCNICA 36/2021-SECOVID/GAB/SECOVID/MS-** Inclusion of children and adolescents between 12 and 17 years old in the target audience for vaccination against Covid-19	- Vaccination against Covid-19 for the population aged 12 to 17 years with and without comorbidities, starting on September 15, 2021 and exclusively with the Comirnaty-Pfizer/Wyeth, according to the following order of priority:<br><br>a) Population aged 12 to 17 with permanent disabilities.<br>b) Population between 12 and 17 years old with presence of comorbidities<br>c) Population between 12 and 17 years old, pregnant and postpartum women<br>d) Population between 12 and 17 years of age deprived of liberty<br>e) Population aged 12 to 17 years without comorbidity
15/09/2021	**NOTA TÉCNICA Nº 40/2021-SECOVID/GAB/SECOVID/MS-**Temporary suspension of vaccination of adolescents without comorbidities	- Vaccination against Covid-19 for the population aged 12 to 17 years with comorbidities or population deprived of liberty, starting on September 15, 2021 and exclusively with the Comirnaty-Pfizer/Wyeth, according to the following order of priority:<br><br>a) Pregnant, postpartum and lactating population, with or without comorbidity, regardless of the age of the infants;<br>b) Population aged between 12 and 17 with permanent disabilities;<br>c) Population between 12 and 17 years old with presence of comorbidities;<br>d) Population aged 12 to 17 years deprived of liberty
15/09/2021	**Nota Técnica Nº 1/2021-SECOVID/GAB/SECOVID/MS -** Review of the recommendation for immunization against COVID-19 in adolescents (NOTA TÉCNICA Nº 40/2021-SECOVID/GAB/SECOVID/MS)	- Vaccination against COVID-19 in adolescents, restricting their employment only to 12 to 17 year olds who have a permanent disability, comorbidities or are deprived of their liberty.
22/09/2021	**NOTA TÉCNICA Nº 45/2021 SECOVID/GAB/SECOVID/MS -** Revocation of the NOTA TÉCNICA Nº 40/2021-SECOVID/GAB/SECOVID/MS	- Vaccination for adolescents aged 12 to 17 years without and with comorbidities, with the Comirnaty- Pfizer
09.02.2022	**NOTA TÉCNICA Nº 8/2022-SECOVID/GAB/SECOVID/MS -** Recommendations for the administration of the primary and booster dose of vaccines against COVID-19 in immunocompromised persons from 12 to 17 years of age	- Vaccination (Primary and booster) for immunocompromised adolescents aged 12 to 17, with the Comirnaty- Pfizer:<br><br>a) Primary dose: Pfizer - Second dose: 8 weeks after the primary dose; Additional dose: 08 weeks after second dose; Booster: 4 months after the additional dose
21.01.2022 - ANVISA Authorization for the Coronavac vaccine in adolescents 12 to 17 years of age , provided that such groups are not immunosuppressed		

21.01.2022	**NOTA TÉCNICA Nº 6/2022-SECOVID/GAB/SECOVID/MS -** Authorization to vaccinate children aged 6 and over and adolescents up to 17 years of age with the Coronavac	- Vaccination of children aged 6 and over and adolescents up to 17 years of age with the Coronavac, provided that such groups are not immunosuppressed
23.02.2022	**NOTA TÉCNICA Nº 11/2022-SECOVID/GAB/SECOVID/MS -** Consolidation of the Technical Notes referring to vaccination of the population over 12 years of age	- Adolescents aged 12 to 17 anos, no pregnancy or puerperal, without comorbidities:<br><br>a) Primary dose: Pfizer , Second dose: 8 weeks after the primary dose<br>b) Primary dose: Coronavac , Second dose: 4 weeks after the primary dose<br>No additional dose or booster indicated<br><br>-Pregnancy or puerperal adolescents aged 12 to 17 anos,without comorbidities:<br><br>a) Primary dose: Pfizer - Second dose: 8 weeks after the primary dose, Booster: 4 months after second dose (preferably Pfizer)<br>b) Primary dose: Coronavac - Second dose: 4 weeks after the primary dose,pregnancy or puerperal (preferably Pfizer)<br>No additional dose indicated<br><br>- Immunocompromised adolescents aged 12 to 17 years, including immunocompromised pregnancy and puerperal:<br><br>a) Primary dose: Pfizer- Second dose: 8 weeks after the primary dose; Aditional dose:8 weeks after the second dose; Booster: 4 months after the aditional dose

Table S2. Vaccination plan for adolescents aged 12 to 17 years in Scotland.

**Date**	**Technical notes issued by the UK or Scottish Government**	**Recommendations**
09/07/2021 - The Conditional Marketing Authorisation for Pfizer-BioNTech BNT162b2 came into effect on 9 July 2021, with approval previously being provided under Regulation 174. JCVI advises that only UK authorised COVID-19 vaccines should be offered to those aged less than 18 years. At this time, the Pfizer-BioNTech BNT162b2 vaccine is the only vaccine authorised for persons aged 12 to 17 years in the UK.		
06/08/2021	1.https://www.gov.scot/news/vaccinations-for-16-to-17-year-olds/<br><br>2.https://www.gov.uk/government/publications/jcvi-statement-august-2021-covid-19-vaccination-of-children-and-young-people-aged-12-to-17-years/jcvi-statement-on-covid-19-vaccination-of-children-and-young-people-aged-12-to-17-years-4-august-2021<br><br>3.https://www.nhsinform.scot/covid-19-vaccine/the-vaccines/vaccinating-children-	**VACCINATION FOR 16 TO 17-YEAR-OLDS**<br><br>All young people 16 to 17 years of age will now be offered the coronavirus (COVID-19) vaccination in Scotland. In line with the latest advice from the Joint Committee on Vaccination and Immunisation (JCVI), they will be offered a first dose of the Pfizer-BioNTech vaccine. From **Friday 6 August**, people who are 16 or 17 in mainland Scotland will be invited to register their interest through the online portal at NHS Inform, and will then be sent an appointment via SMS or email. Eligible young people in Shetland, Orkney and Western Isles will be contacted by their health board and invited to attend clinics.<br><br>Alternatively, drop-in clinics will be available for 16 to 17-years-olds. The start date for clinics opening for this age group will be confirmed shortly. Anyone who doesn't register an interest or attend a drop-in clinic, once open, will be sent an appointment invitation through the post. It is expected that everyone in this age

	and-young-people-aged-12-to-17-years/<br><br>4.https://www.gov.uk/government/publications/regulatory-approval-of-pfizer-biontech-vaccine-for-covid-19/summary-of-product-characteristics-for-covid-19-vaccine-pfizerbiontech	group will have been offered a vaccination appointment by the end of September.<br><br>**ADDITIONAL AT-RISK GROUPS OF MINORS**<br><br>In addition to 16 to 17-year-olds, we have started offering COVID-19 vaccinations to children and young people who are from key groups; and are between 12 to 17 years. Vaccinations will be offered to:<br><br>12 to 15-year-olds with severe neuro-disabilities<br><br>12 to 15-year-olds with Down's syndrome<br><br>12 to 15-year-olds with underlying conditions resulting in immunosuppression<br><br>12 to 15-year-olds with profound and multiple learning disabilities (PMLD)<br><br>12 to 15-year-olds with severe learning disabilities<br><br>12 to 17-year-olds who have a diagnosed learning/intellectual disability (mild or moderate)<br><br>16-year-olds who have any of these above conditions or underlying conditions that place them at higher risk of serious COVID-19 (that were not 16 at the time of the previous invite for all 16 and 17-year-olds in March 2021)<br><br>young people aged 12 years and above who live in the same household of persons (adults or children) who are immunosuppressed (we previously invited over 16s who are household contacts of those on the shielding list)
20/09/2021	1.https://www.gov.scot/news/vaccinations-for-12-15-year-olds/<br><br>2.https://www.gov.scot/publications/coronavirus-covid-19-update-first-ministers-statement-14-september-2021/<br><br>3.https://www.nhsinform.scot/covid-19-vaccine/the-vaccines/vaccinating-children-and-young-people-aged-12-to-17-years/	**VACCINATION FOR 12 TO 15-YEAR-OLDS**<br><br>Children and young people aged 12 -15 years old will be offered a dose of the coronavirus (COVID-19) vaccination from **Monday 20 September** after Scottish Ministers accepted advice from the four UK Chief Medical Officers (CMOs). As a result, a dose of Pfizer-BioNTech vaccine will be offered to all children and young people aged 12-15 who are not already covered by existing advice from the Joint Committee on Vaccination and Immunisation (JCVI) in a move to reduce the disruption caused to education by COVID-19. This group will be offered their injections in drop-in clinics and community settings followed by each young person receiving a letter inviting them to attend a community clinic. For some rural Health Boards, those aged 12 to 15 will first be offered the vaccine at school.<br><br>Following the initial phase, vaccines will be offered in both communities and schools so that anyone who hasn't been vaccinated but would like to be has the opportunity to take up the offer. Meanwhile, people who received their vaccination during phase one of the national COVID-19 vaccination programme in Scotland will start to receive booster injections **from 20 September**. This follows advice from the JCVI which has advised that the booster dose can be given alongside the flu jab and should be offered no earlier than six months after completion of the

		primary vaccine course. Frontline health and social care workers will be able to book their appointment online at NHS Inform from 20 September and from that date, residents in care homes for older people will be offered both flu and COVID-19 booster vaccination.<br><br>Children aged 12-15 who have specific underlying conditions or disabilities are already covered by previous JCVI advice and will be offered two doses, eight weeks apart.<br><br>**DETAILS OF PROCEDURES**<br><br>NHS Scotland is offering 2 doses of the coronavirus vaccine to all children and young people aged 12 to 17 years. All children and young people aged 16 and 17 years are eligible for a booster dose. Some children and young people at higher risk from coronavirus are also eligible for additional doses (third primary dose and/or booster doses).<br><br>Children and young people aged 12 to 17 years, who have recently tested positive for coronavirus, should wait 12 weeks after the date they were tested to get the vaccine (any dose). However, those aged 12 to 17 and at higher risk from coronavirus, can have your coronavirus vaccine from 4 weeks after coronavirus infection.<br><br>Children and young people aged 12 to 17 years were offered a second dose of the vaccine from 12 weeks after the first dose. All young people aged 16 or 17 years were eligible for a booster dose. Some children and young people aged 12 to 15 years at higher risk from coronavirus were eligible for a booster dose, from 12 weeks after their second dose. This includes those: (i) who are at increased risk from coronavirus due to underlying health conditions; (ii) who share living accommodation, on most days, with someone who has a weakened immune system; (iii) with a severely weakened immune system who have had a third primary dose.<br><br>Children and young people aged 12 to 17 years with a weakened immune system were also offered a spring booster dose of the coronavirus vaccine. The spring booster dose was usually offered around 6 months (and not before 3 months) since the last dose of the coronavirus vaccine. Children and young people aged 12 to 17 years who were at increased risk from coronavirus were offered 2 doses of the vaccine, given 8 weeks apart. This included those who: (i) were at increased risk from coronavirus due to underlying health conditions; (ii) shared living accommodation, on most days, with someone who has a weakened immune system; (iii) were aged 16 or 17 years who were an unpaid carer or a frontline health or social care worker.<br><br>Children and young people aged 12 to 17 years with a severely weakened immune system were offered 3 primary doses of the coronavirus vaccine. They were also eligible for a booster dose to help improve protection.

Table S3. Checklist: The RECORD statement[1]

	Item No.	STROBE items	RECORD items	Location in manuscript where items are reported
	1	(a) Indicate the study's design with a commonly used term in the title or the abstract (b) Provide in the abstract an informative and balanced summary of what was done and what was found	RECORD 1.1: The type of data used should be specified in the title or abstract. When possible, the name of the databases used should be included.<br><br>RECORD 1.2: If applicable, the geographic region and timeframe within which the study took place should be reported in the title or abstract.<br><br>RECORD 1.3: If linkage between databases was conducted for the study, this should be clearly stated in the title or abstract.	Title: *"Two-dose BNT162b2 vaccine effectiveness against COVID-19 symptomatic infection and severe cases among adolescents: A test negative design in Brazil and Scotland".*<br><br>The abstract has the requested information
Background rationale	2	Explain the scientific background and rationale for the investigation being reported		Paragraph 2 and 3
Objectives	3	State specific objectives, including any prespecified hypotheses		Paragraph 4
Study Design	4	Present key elements of study design early in the paper		Paragraph 1 of the Methods section.
Setting	5	Describe the setting, locations, and relevant dates, including periods of recruitment, exposure, follow-up, and data collection		Paragraph 1 and 2 of the Methods section.
Participants	6	*(a) Cohort study* - Give the eligibility criteria, and the sources and methods of selection of participants. Describe methods of follow-up<br>*Case-control study* - Give the eligibility criteria, and the sources and methods of case ascertainment and control selection. Give the rationale for the choice of cases and controls	RECORD 6.1: The methods of study population selection (such as codes or algorithms used to identify subjects) should be listed in detail. If this is not possible, an explanation should be provided.<br><br>RECORD 6.2: Any validation studies of the codes or algorithms used to select the population should be referenced. If validation was conducted for this study and not published elsewhere, detailed methods and results should be provided.	The data source and linkage process are described in the methods section, including references that describe the linkage algorithm.<br>Paragraphs 2 and 3 and Figure 1.

		Cross-sectional study - Give the eligibility criteria, and the sources and methods of selection of participants<br><br>*(b) Cohort study* - For matched studies, give matching criteria and number of exposed and unexposed<br>*Case-control study* - For matched studies, give matching criteria and the number of controls per case	RECORD 6.3: If the study involved linkage of databases, consider use of a flow diagram or other graphical display to demonstrate the data linkage process, including the number of individuals with linked data at each stage.	
Variables	7	Clearly define all outcomes, exposures, predictors, potential confounders, and effect modifiers. Give diagnostic criteria, if applicable.	RECORD 7.1: A complete list of codes and algorithms used to classify exposures, outcomes, confounders, and effect modifiers should be provided. If these cannot be reported, an explanation should be provided.	Paragraph 5 and 6
Data sources/ measurement	8	For each variable of interest, give sources of data and details of methods of assessment (measurement).<br>Describe comparability of assessment methods if there is more than one group		Paragraph 3 and Supplementary material (Detailed information of Brazilian Database)
Bias	9	Describe any efforts to address potential sources of bias		Paragraph 2<br>Paragraph 5 of Results
Study size	10	Explain how the study size was arrived at		Figure 1
Quantitative variables	11	Explain how quantitative variables were handled in the analyses. If applicable, describe which groupings were chosen, and why		Paragraph 5 and 6, and Table 1.
Statistical methods	12	(a) Describe all statistical methods, including those used to control for confounding<br>(b) Describe any methods used to examine subgroups and interactions<br>(c) Explain how missing data were addressed<br>(d) *Cohort study* - If applicable, explain how loss to follow-up was addressed		Paragraph 7

		Case-control study - If applicable, explain how matching of cases and controls was addressed<br>*Cross-sectional study* - If applicable, describe analytical methods taking account of sampling strategy<br>(e) Describe any sensitivity analyses		
Data access and cleaning methods		..	RECORD 12.1: Authors should describe the extent to which the investigators had access to the database population used to create the study population.<br><br>RECORD 12.2: Authors should provide information on the data cleaning methods used in the study.	Paragraph 3 and Supplementary appendix (Detailed information of Brazilian Database)
Linkage		..	RECORD 12.3: State whether the study included person-level, institutional-level, or other data linkage across two or more databases. The methods of linkage and methods of linkage quality evaluation should be provided.	Paragraph 3
Participants	13	(a) Report the numbers of individuals at each stage of the study (*e.g.*, numbers potentially eligible, examined for eligibility, confirmed eligible, included in the study, completing follow-up, and analysed)<br>(b) Give reasons for non-participation at each stage.<br>(c) Consider use of a flow diagram	RECORD 13.1: Describe in detail the selection of the persons included in the study (*i.e.*, study population selection) including filtering based on data quality, data availability and linkage. The selection of included persons can be described in the text and/or by means of the study flow diagram.	Paragraph 1 of the Result section.
Descriptive data	14	(a) Give characteristics of study participants (*e.g.*, demographic, clinical, social) and information on exposures and potential confounders<br>(b) Indicate the number of participants with missing data for each variable of interest<br>(c) *Cohort study* - summarise follow-up time (*e.g.*, average and total amount)		Paragraph 1
Outcome data	15	*Cohort study* - Report numbers of outcome events or summary measures over time		Table 1, 2, 3 and 4, Figures 2 and 3

		Case-control study - Report numbers in each exposure category, or summary measures of exposure<br>*Cross-sectional study* - Report numbers of outcome events or summary measures		
Main results	16	(a) Give unadjusted estimates and, if applicable, confounder-adjusted estimates and their precision (e.g., 95% confidence interval). Make clear which confounders were adjusted for and why they were included<br>(b) Report category boundaries when continuous variables were categorized<br>(c) If relevant, consider translating estimates of relative risk into absolute risk for a meaningful time period		Paragraphs 2-6, Table 2-4, Figures 2 and 3
Other analyses	17	Report other analyses done—e.g., analyses of subgroups and interactions, and sensitivity analyses		Paragraph 2 and Supplemetary material
Key results	18	Summarise key results with reference to study objectives		Paragraph 1 of the discussion section.
Limitations	19	Discuss limitations of the study, taking into account sources of potential bias or imprecision. Discuss both direction and magnitude of any potential bias	RECORD 19.1: Discuss the implications of using data that were not created or collected to answer the specific research question(s). Include discussion of misclassification bias, unmeasured confounding, missing data, and changing eligibility over time, as they pertain to the study being reported.	Paragraph 6
Interpretation	20	Give a cautious overall interpretation of results considering objectives, limitations, multiplicity of analyses, results from similar studies, and other relevant evidence		Paragraph 7
Generalisability	21	Discuss the generalisability (external validity) of the study results		Paragraph 6
Funding	22	Give the source of funding and the role of the funders for the present study and, if		We included a statement about the funding role.

	applicable, for the original study on which the present article is based		
Accessibility of protocol, raw data, and programming code	..	RECORD 22.1: Authors should provide information on how to access any supplemental information such as the study protocol, raw data, or programming code.	We included a section on data availability.

Table S4. Estimates for covariates in logistic regression model for Omicron and Delta in Brazil

Factor	Level	Omicron		Delta		Omicron (Severe cases)	
Week*		OR	95% CI	OR	95% CI	OR	95% CI
	1	1·00	Baseline	1·00	Baseline	1·00	Baseline
	2	1·74	(1·68,1·79)	1·21	(1·11,1·31)	1·96	(1·28,3·00)
	3	2·47	(2·39,2·55)	1·02	(0·94,1·10)	3·49	(2·33,5·22)
	4	2·72	(2·63,2·81)	0·87	(0·80,0·94)	4·38	(2·93,6·53)
	5	2·70	(2·61,2·79)	0·76	(0·70,0·82)	4·58	(3·02,6·93)
	6	2·17	(2·09,2·24)	0·73	(0·67,0·80)	3·64	(2·35,5·63)
	7	1·58	(1·52,1·64)	0·87	(0·79,0·95)	2·07	(1·27,3·38)
	8	1·00	(0·96,1·04)	0·64	(0·58,0·70)	1·14	(0·63,2·04)
	9	0·64	(0·61,0·68)	0·58	(0·53,0·63)	1·20	(0·60,2·42)
	10	0·37	(0·35,0·39)	0·66	(0·60,0·73)	0·91	(0·46,1·79)
	11	0·25	(0·24,0·27)	0·55	(0·50,0·61)	0·90	(0·43,1·86)
	12	0·18	(0·17,0·19)	0·57	(0·51,0·62)	0·29	(0·10,0·85)
	13	0·16	(0·15,0·18)	0·52	(0·47,0·57)	0·28	(0·08,0·93)
	14	0·18	(0·16,0·19)	0·49	(0·44,0·54)	0·60	(0·23,1·57)
	15	0·20	(0·19,0·22)	0·44	(0·40,0·49)	0·34	(0·07,1·56)
	16	0·22	(0·18,0·26)	0·43	(0·38,0·48)	0·00	(0·00,1·13eˆ283)
	17	0·50	(0·41,0·62)	0·38	(0·34,0·43)	2·69	(0·63,11·41)
	18			0·82	(0·74,0·90)		
**Gender**	F	1·00	Baseline	1·00	Baseline	1·00	Baseline
	M	0·95	(0·93,0·96)	0·99	(0·96,1·01)	1·11	(0·92,1·32)
**Age**	12	1·00	Baseline	1·00	Baseline	1·00	Baseline
	13	1·01	(0·98,1·04)	1·09	(1·03,1·15)	1·26	(0·88,1·81)
	14	1·07	(1·04,1·09)	1·15	(1·09,1·21)	1·75	(1·25,2·45)
	15	1·07	(1·04,1·09)	1·18	(1·12,1·24)	1·95	(1·41,2·69)
	16	1·06	(1·03,1·09)	1·21	(1·15,1·28)	1·98	(1·45,2·72)
	17	1·04	(1·01,1·06)	1·18	(1·12,1·24)	1·81	(1·32,2·48)
**Ethnicity**	White (1)	1·00	Baseline	1·00	Baseline	1·00	Baseline
	Black (2)	0·73	(0·70,0·77)	0·79	(0·72,0·86)	0·56	(0·32,0·97)
	Asian (3)	0·81	(0·76,0·86)	0·92	(0·80,1·06)	0·57	(0·25,1·30)
	Mixed (4)	0·80	(0·79,0·82)	0·86	(0·83,0·90)	0·69	(0·55,0·87)
	Indigenous (5)	0·50	(0·44,0·58)	1·85	(1·52,2·25)	0·42	(0·10,1·78)
	Missing (6)	1·03	(1·01,1·05)	1·08	(1·04,1·13)	0·63	(0·48,0·82)
**Deprivation Index**	1 (least)	1·00	Baseline	1·00	Baseline	1·00	Baseline
	2	1·09	(1·07,1·12)	1·24	(1·19,1·30)	1·57	(1·20,2·04)
	3	1·19	(1·16,1·22)	1·24	(1·18,1·30)	1·62	(1·23,2·12)
	4	1·51	(1·47,1·55)	1·40	(1·32,1·47)	1·62	(1·17,2·26)
	5 (most)	1·35	(1·30,1·40)	1·71	(1·59,1·83)	1·09	(0·71,1·66)
**Comorbidities**	0	1·00	Baseline	1·00	Baseline	1·00	Baseline
	1	0·78	(0·75,0·81)	0·84	(0·77,0·90)	6·74	(5·38,8·45)
	2+	1·07	(0·85,1·36)	0·86	(0·58,1·29)	26·16	(14·29,47·88)
**Previous confirmed infection**	0	1·00	Baseline	1·00	Baseline	1·00	Baseline
	3-6 months	0·48	(0·43,0·54)	0·20	(0·15,0·25)	0·00	(0·00,5·47eˆ225)
	6+ months	0·51	(0·49,0·53)	0·19	(0·16,0·23)	0·59	(0·34,1·03)
**Pregnancy**	No (0)	1·00	Baseline	1·00	Baseline	1·00	Baseline
	Yes (1)	1·07	(0·94,1·21)	0·45	(0·36,0·58)	19·54	(13·67,27·93)
**Post-partum**	No (0)	1·00	Baseline	1·00	Baseline	1·00	Baseline
	Yes (1)	0·72	(0·48,1·10)	0·89	(0·47,1·67)	46·82	(24·07,91·05)
**States**	São Paulo (35)	1·00	Baseline	1·00	Baseline	1·00	Baseline
	Rondônia (11)	1·12	(1·04,1·20)	3·28	(2·92,3·68)	0·14	(0·02,1·00)
	Acre (12)	0·88	(0·72,1·07)	1·40	(0·56,3·46)	0·69	(0·09,5·41)

Amazonas (13)	0·72	(0·65,0·79)	1·19	(0·98,1·45)	1·81	(0·90,3·62)
Roraima (14)	0·63	(0·53,0·74)	0·73	(0·54,0·97)	0·00	(0·00 ,Inf)
Pará (15)	0·80	(0·73,0·86)	3·19	(2·84,3·60)	3·16	(1·76,5·70)
Amapá (16)	1·27	(1·00,1·60)	1·98	(1·58,2·49)	0·00	(0·00,Inf)
Tocantins (17)	0·73	(0·67,0·79)	3·02	(2·64,3·45)	0·69	(0·24,1·99)
Maranhão (21)	0·77	(0·69,0·86)	2·53	(2·04,3·14)	0·20	(0·03,1·52)
Piauí (22)	1·08	(0·99,1·17)	4·15	(3·68,4·69)	2·46	(1·11,5·46)
Ceará (23)	0·46	(0·44,0·49)	0·97	(0·85,1·10)	1·37	(0·82,2·28)
Rio Grande do Norte (24)	0·65	(0·61,0·69)	1·13	(1·00,1·28)	0·13	(0·03,0·55)
Paraíba (25)	0·82	(0·77,0·87)	2·11	(1·85,2·41)	1·15	(0·60,2·21)
Pernambuco (26)	0·59	(0·57,0·63)	0·87	(0·79,0·97)	0·24	(0·10,0·63)
Alagoas (27)	0·68	(0·61,0·76)	0·55	(0·40,0·75)	1·71	(0·65,4·51)
Sergipe (28)	0·74	(0·68,0·82)	0·42	(0·31,0·59)	1·72	(0·68,4·37)
Bahia (29)	0·91	(0·86,0·96)	1·84	(1·68,2·01)	1·99	(1·23,3·21)
Minas Gerais (31)	1·25	(1·21,1·28)	1·96	(1·86,2·07)	1·17	(0·86,1·58)
Espírito Santos (32)	0·58	(0·37,0·92)	0·90	(0·40,2·02)	14·93	(4·92,45·29)
Rio de Janeiro (33)	0·63	(0·61,0·65)	0·89	(0·83,0·95)	0·47	(0·30,0·75)
Paraná (41)	1·36	(1·17,1·59)	1·66	(1·35,2·05)	26·89	(18·41,39·27)
Santa Catarina (42)	1·10	(1·07,1·13)	1·28	(1·21,1·35)	0·81	(0·58,1·13)
Rio Grande do Sul (43)	1·10	(1·07,1·12)	1·22	(1·16,1·29)	0·38	(0·26,0·55)
Mato Grosso do Sul (50)	0·71	(0·67,0·76)	0·66	(0·55,0·80)	0·92	(0·51,1·64)
Mato Grosso (51)	1·10	(1·04,1·16)	2·41	(2·18,2·67)	0·88	(0·46,1·68)
Goiás (52)	0·89	(0·86,0·93)	2·67	(2·48,2·87)	0·39	(0·22,0·68)
Distrito Federal (53)	0·96	(0·89,1·03)	2·82	(3·24,14·62)	0·14	(0·02,1·04)

***OR: Odds Ratio; **We used epidemiological week to adjust the analyses for Omicron period we used 52-16 week and for Delta 36-52 week.**

Table S5. Estimates for covariates in logistic regression model for Omicron and Delta in Scotland.

Factor	Level	Omicron		Delta	
Week*		OR	95% CI	OR	95% CI
	1	1·00	Baseline	1·00	Baseline
	2	1·14	(1·04,1·24)	0·88	(0·68,1·15)
	3	0·78	(0·71,0·86)	0·83	(0·65,1·05)
	4	0·70	(0·63,0·77)	0·68	(0·54,0·86)
	5	0·76	(0·68,0·83)	0·47	(0·37,0·59)
	6	0·61	(0·55,0·68)	0·38	(0·30,0·48)
	7	0·58	(0·51,0·64)	0·36	(0·29,0·46)
	8	0·90	(0·79,1·01)	0·35	(0·28,0·45)
	9	0·84	(0·74,0·94)	0·33	(0·26,0·41)
	10	0·72	(0·64,0·81)	0·43	(0·34,0·54)
	11	0·85	(0·77,0·95)	0·69	(0·54,0·88)
	12	0·75	(0·67,0·82)	0·48	(0·37,0·61)
	13	0·66	(0·59,0·73)	0·39	(0·31,0·50)
	14	0·64	(0·57,0·72)	0·41	(0·32,0·52)
	15	0·77	(0·66,0·89)	0·46	(0·36,0·59)
	16	0·83	(0·70,1·00)	0·45	(0·35,0·57)

	17	0·80	(0·65,0·98)	0·50	(0·39,0·63)
	18			0·53	(0·42,0·67)
	19			0·52	(0·41,0·67)
	20			0·76	(0·60,0·96)
	21			1·20	(0·91,1·58)
**Gender**	F	1·00	Baseline	1·00	Baseline
	M	0·91	(0·87,0·95)	0·95	(0·92,0·98)
**Age**	12	1·00	Baseline	1·00	Baseline
	13	1·04	(0·97,1·10)	0·99	(0·95,1·04)
	14	1·10	(1·03,1·18)	1·05	(1·01,1·11)
	15	1·19	(1·10,1·27)	1·14	(1·09,1·19)
	16	1·34	(1·25,1·43)	1·25	(1·19,1·32)
	17	1·67	(1·55,1·79)	1·44	(1·36,1·54)
**Deprivation Index**	1 (least)	1·00	Baseline	1·00	Baseline
	2	0·99	(0·93,1·06)	1·03	(0·98,1·08)
	3	0·93	(0·87,0·99)	0·99	(0·95,1·04)
	4	0·89	(0·84,0·95)	0·98	(0·94,1·03)
	5 (most)	0·90	(0·84,0·96)	0·96	(0·92,1·00)
**Comorbidities (Q-Covid conditions)**	0	1·00	Baseline	1·00	Baseline
	Asthma	0·89	(0·84,0·94)	0·80	(0·77,0·84)
	Blood Cancer	0·72	(0·34,1·51)	0·64	(0·29,1·40)
	Cerebral Palsy	0·68	(0·40,1·169)	0·97	(0·62,1·50)
	Congenital heart defect	1·02	(0·80,1·31)	0·99	(0·83,1·18)
	Epilepsy	0·93	(0·72,1·21)	0·94	(0·77,1·15)
	Fracture	0·93	(0·83,1·04)	1·07	(0·99,1·16)
	Severe Mental Illness	0·70	(0·43,1·13)	0·62	(0·42,0·91)
	Learning difficulties	0·94	(0·85,1·04)	0·77	(0·71,0·83)
**Previous confirmed infection**	0	1·00	Baseline	1·00	Baseline
	3-6 months	0·35	(0·27,0·44)	0·08	(0·06,0·10)
	6+ months	0·46	(0·42,0·50)	0·11	(0·09,0·13)
**Number of test/person**	0	1·00	Baseline	1·00	Baseline
	1	0·81	(0·76,0·86)	0·92	(0·88,0·95)
	2	0·70	(0·66,0·75)	0·90	(0·86,0·94)
	3	0·57	(0·54,0·62)	0·84	(0·79,0·89)
	4	0·58	(0·53,0·63)	0·81	(0·75,0·89)
	5 to 9	0·51	(0·47,0·56)	0·73	(0·66,0·81)
	10 to 19	0·44	(0·33,0·59)	0·71	(0·42,1·18)
	20+	1·34	(0·57,3·12)	0·88	(0·26,2·98)
**Regions**	Large Urban Areas	1·00	Baseline	1·00	Baseline
	Other Urban Areas	0·92	(0·88,0·97)	0·96	(0·93,1·00)
	Accessible Small Towns	0·85	(0·79,0·92)	0·91	(0·86,0·95)
	Remote Small Towns	0·77	(0·70,0·86)	0·63	(0·58,0·68)
	Accessible Rural	0·91	(0·84,0·98)	0·84	(0·79,0·89)
	Remote Rural	0·79	(0·71,0·87)	0·59	(0·54,0·64)

***OR: Odds Ratio**

Supplemental table 6. Table of characteristic during the Delta period for Brazil and Scotland

Characteristic	Brazil Cases n = 25,711	Brazil Controls n = 122,999	Scotland Cases n = 34,397	Scotland Controls n = 47,022
**Age**				
12	3,345 (13·0%)	16,662 (13·5%)	6,289 (18.3%)	8,821 (18.8%)
13	3,496 (13·6%)	17,848 (14·5%)	6,605 (19.2%)	9,393 (20.0%)
14	3,944 (15·3%)	19,320 (15·7%)	6,510 (18.9%)	8,890 (18.9%)
15	4,543 (17·7%)	21,556 (17·5%)	6,380 (18.5%)	8,026 (17.1%)
16	5,025 (19·5%)	22,680 (18·4%)	5,451 (15.8%)	7,706 (16.4%)
17	5,358 (20·8%)	24,933 (20·3%)	3,162 (9.2%)	4,186 (8.9%)
**Sex**				
Female	13,504 (52·5%)	64,191 (52·2%)	17,391 (50.6%)	23,275 (49.5%)
Male	12,207 (47·5%)	58,808 (47·8%)	17,006 (49.4%)	23,747 (50.5%)
**Deprivation Index (quintile)**				
1	6,929 (26·9%)	47,766 (38·8%)	7,824 (22.7%)	9,974 (21.2%)
2	4,956 (19·3%)	25,175 (20·5%)	7,050 (20.5%)	9,098 (19.3%)
3	4,329 (16·8%)	19,325 (15·7%)	5,921 (17.2%)	8,475 (18.0%)
4	5,483 (21·3%)	18,511 (15·0%)	6,590 (19.2%)	9,418 (20.0%)
5	4,014 (15·6%)	12,222 (9·9%)	7,012 (20.4%)	10,057 (21.4%)
**Number of comorbidities**				
0	24,889 (96·8%)	117,894 (95·8%)	28,352 (82.4%)	37,265 (79.3%)
1	791 (3·1%)	4,934 (4·0%)	5,549 (16.1%)	8,807 (18.7%)
>=2	31 (0·1%)	171 (0·1%)	496 (1.4%)	950 (2.0%)
**Previous confirmed infection**				
No	25,524 (99·3%)	118,086 (96·0%)	34,147 (99.3%)	44,071 (93.7%)
3-6 month	67 (0·3%)	1,677 (1·4%)	66 (0.2%)	1,040 (2.2%)
>6 month	120 (0·5%)	3,236 (2·6%)	184 (0.5%)	1,911 (4.1%)
**Hospitalization**				
No event	25,420 (98·9%)	121,934 (99·1%)	34,268 (99.6%)	46,970 (99.9%)
Yes	291 (1·1%)	1,065 (0·9%)	129 (0.4%)	52 (0.1%)
**Death**				
No event	25,690 (99·9%)	122,934 (99·9%)	34,396 (100.0%)	47,022 (100.0%)
Yes	21 (0·1%)	65 (0·1%)	1 (0.0%)	0 (0.0%)

Table S7. Specific demographic for Brazil in Omicron and Delta Period

**Brazil**	Omicron		Delta	
**Ethnicity**	**Case**	**Controls**	**Case**	**Controls**
White	69,142 (46·0%)	91,909 (44·9%)	10,819 (42·1%)	56,665 (46·1%)
Black	3,796 (2·5%)	6,608 (3·2%)	679 (2·6%)	4,236 (3·4%)
Asian	2,066 (1·4%)	2,957 (1·4%)	295 (1·1%)	1,159 (0·9%)
Mixed	44,828 (29·8%)	66,314 (32·4%)	8,658 (33·7%)	38,832 (31·6%)
Indigenous	319 (0·2%)	693 (0·3%)	190 (0·7%)	380 (0·3%)
Missing*	30,140 (20·1%)	36,294 (17·7%)	5,070 (19·7%)	21,727 (17·7%)
**Pregnancy**				
No	149,769 (99·7%)	204,180 (99·7%)	25,634 (99·7%)	122,393 (99·5%)
Yes	522 (0·3%)	595 (0·3%)	77 (0·3%)	606 (0·5%)
**Post-partum**				
No	150,250 (100·0%)	204,714 (100·0%)	25,698 (99·9%)	122,940 (100·0%)
Yes	41 (0·0%)	61 (0·0%)	13 (0·1%)	59 (0·0%)

***The missing data were included to the analyses as level from ethnicity.**

Table S8. Vaccine effectiveness based on multiple imputations analyses.

BRAZIL	VE (%) (95% CI)		
	Omicron		Delta
Vaccination Status	Symptomatic Infection	Severe Outcomes	Symptomatic Infection
Unvaccinated			
**1st dose**			
0-6 days	37·1 (28·1,45·0)	19·0 (-156·9,74·5)	9·1 (1·9,15·8)
7-13 days	20·1 (11·1,28·3)	62·6 (-21.8,88·5)	4·1 (-1·8,9·7)
>=14-2nd dose	28·1 (26·3,29·8)	56·2 (45·8,64·6)	52·5 (50·6,54·3)
**2nd dose**			
0-13 days	58·8 (56·4,61·0)	64·8 (36·9,80·4)	71·7 (68·0,74·9)
14-27 days	64·8 (63·1,66·4)	75·6 (58·1,85·8)	80·8 (77·8,83·3)
28-41 days	53·1 (51·4,54·7)	82·6 (71·8,89·3)	68·2 (63·3,72·4)
42-55 days	40·8 (38·9,42·5)	84·0 (76·0,89·4)	37·8 (27·2,46·9)
56-69 days	32·2 (30·1,34·1)	83·5 (75·9,88·8)	27·0 (4·5,44·1)
70-83 days	25·6 (23·2,28·0)	82·0 (72·4,88·2)	
84-97 days	17·4 (14·4,20·4)	86·4 (75·2,92·5)	
>=98 days	6·5 (2·9,10·0)	82·7 (68·8,90·4)	

Figure S1. VE from severe cases (hospitalisation or death) by the length of time since the first and second doses of BNT162b2 in Brazil during the Omicron dominant period.

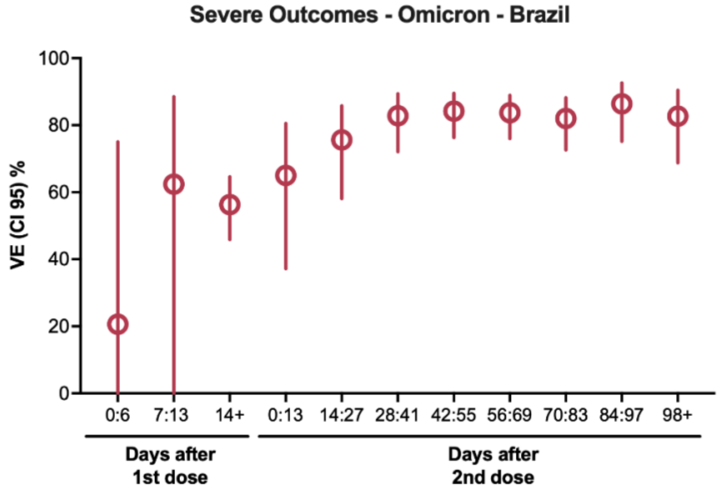

Supplemental methods

Detailed information of Brazilian Database

The **e-SUS Notifica**: This database contains information on all suspected cases of Covid-19 recorded in the country. It includes all positive and negative RT-PCR test results, and information on residence, demographic and clinical data of individuals, such as the presence of comorbidities and pregnancy status and presence of symptoms, with acute respiratory diseases defined as the presence of at least two of the following signs and symptoms: fever (even if

referred), chills, sore throat, headache, cough, runny nose, loss or change to a sense of smell or taste.[2] Asymptomatic individuals with an RT-PCR test were not included in this study, independent of the test result. This database has been used as a data source for epidemiological research. [3]

The **SIVEP-Gripe** is the national registration for severe acute respiratory syndrome (SARS) in Brazil, created after the Influenza pandemic of 2009. In 2020, it was expanded to include Covid-19. All Covid-19 hospitalisations and deaths are meant to be registered in this system. In this system, severe acute respiratory illness is defined as an individual who presents dyspnea/respiratory discomfort, persistent pressure or pain in the chest, oxygen saturation less than 95% without oxygen, or cyanosis of the lips or face.[2] Also, children registered may experience air loss, dehydration, or loss of appetite. Individuals who died with severe acute respiratory illness independent of hospitalisation are also registered. This database has also been widely used as a source for epidemiological studies.[4,5]

The **SI-PNI** contains data on all vaccines administered in Brazil. Covid-19 vaccines are administered by health services and recorded in point-of-care applications. From SI-PNI, we extracted information on which Covid-19 vaccine was received with first and second doses. By linking these data with the data on adolescents in the other database, we determined: (i) who tested negative for Covid-19 had been vaccinated (ii) with confirmed symptomatic Covid-19 infections had been vaccinated. We assumed that adolescents whose records did not link to an SI-PNI vaccination record were not vaccinated.

List of comorbidities and risk factors

Brazil: Cardiac disease, Diabetes mellitus, Obesity, Immunosuppression, and chronic kidney disease.

Scotland: QCovid conditions - Asthma, Blood Cancer, Cerebral Palsy, Epilepsy, fracture, severe mental illness, learning difficulties or a congenital heart defect.

References

1 Benchimol EI, Smeeth L, Guttmann A, *et al.* The REporting of studies Conducted using Observational Routinely-collected health Data (RECORD) Statement. *PLoS Medicine* 2015; **12**: 1–22.

2 Saiba como é feita a definição de casos suspeitos de Covid-19 no Brasil. Ministério da Saúde. 2021. https://www.gov.br/saude/pt-br/coronavirus/artigos/definicao-e-casos-suspeitos (accessed May 12, 2021).

3 Lima FET, Albuquerque NLS de, Florencio S de SG, *et al.* Time interval between onset of symptoms and COVID-19 testing in Brazilian state capitals, August 2020. *Epidemiologia e servicos de saude : revista do Sistema Unico de Saude do Brasil* 2020; **30**: e2020788.

4 Ranzani OT, Bastos LSL, Gelli JGM, *et al.* Characterisation of the first 250 000 hospital admissions for COVID-19 in Brazil: a retrospective analysis of nationwide data. *The Lancet Respiratory Medicine* 2021; **9**: 407–18.

5 Oliveira EA, Colosimo EA, Simões e Silva AC, *et al.* Clinical characteristics and risk factors for death among hospitalised children and adolescents with COVID-19 in Brazil: an analysis of a nationwide database. *The Lancet Child and Adolescent Health* 2021; **5**: 559–68.
